# Lower Serum Vitamin D Metabolite Levels in Relation to Circulating Cytokines/Chemokines and Metabolic Hormones in Pregnant Women with Hypertensive Disorders

**DOI:** 10.3389/fimmu.2017.00273

**Published:** 2017-03-13

**Authors:** Ramu Adela, Roshan M. Borkar, Navneeta Mishra, Murali Mohan Bhandi, Gayatri Vishwakarma, B. Aparna Varma, Srinivas Ragampeta, Sanjay K. Banerjee

**Affiliations:** ^1^Drug Discovery Research Center, Translational Health Science and Technology Institute (THSTI), Faridabad, Haryana, India; ^2^National Center for Mass Spectrometry, Indian Institute of Chemical Technology (CSIR-IICT), Hyderabad, India; ^3^Department of Biochemistry, Mediciti Institute of Medical Sciences, Ghanpur, Medchal, India; ^4^Clinical Development Service Agency (CDSA), Translational Health Science and Technology Institute (THSTI), Faridabad, Haryana, India

**Keywords:** cytokines, chemokines, metabolic hormones, vitamin D, eclampsia, preeclampsia, gestational hypertension, nutrition

## Abstract

The aim of this study was to investigate whether lower serum vitamin D metabolite levels were associated with altered cytokine/chemokine and metabolic hormone levels in three different hypertensive disorders in pregnancy (HDP). Healthy pregnancy (*n* = 30) and hypertensive disorders in pregnancy (HDP) (*n* = 30), i.e., gestational hypertension (GH), preeclampsia (PE), and eclampsia (EC) subjects were enrolled. Vitamin D metabolites were measured by UPLC/APCI/HRMS method. Circulatory 27 cytokines/chemokines and 10 metabolic hormones were measured. Significantly decreased 25(OH)D and 1,25(OH)_2_D levels were observed in HDP. The levels of 25(OH)D were significantly lower in PE and EC, whereas the serum levels of 1,25(OH)_2_D significantly decreased only in EC subjects. Serum 25(OH)D and 1,25(OH)_2_D levels were negatively correlated with systolic- and diastolic blood pressure, creatinine, and uric acid levels. Serum interleukin (IL)-6 and IL-13 decreased, and GIP levels were increased in gestational hypertensive subjects. Platelet-derived growth factor-BB and IL-8 levels were increased and macrophage inflammatory protein-1beta levels were decreased in EC subjects. IL-8 and IL-10 increased, and rantes and GIP levels decreased in the EC group as compared with the GH group. Multivariate logistic regression analysis showed that eotaxin, monocyte chemotactic protein-1, 25(OH)D, and 1,25(OH)_2_D were predictors of HDP. Our analyses suggest that lower vitamin D metabolites are associated with altered cytokines/chemokines and metabolic hormones in HDP.

## Introduction

Hypertension is the most common medical problem encountered during pregnancy, complicating 5–10% of pregnancies (1). Gestational hypertension (GH), preeclampsia (PE), and eclampsia are spectrum of hypertensive disorders in pregnancy (HDP). Although these disorders can appear in isolation, they are progressive manifestation of a single process and thus share a common etiology (2). HDP is considered as a risk factor for future cardiovascular diseases in later stages of life. Different mechanisms such as endothelial dysfunction, oxidative stress, inflammation, and metabolic abnormalities play important role in HDP (3). Serum biomarkers reflect underlying disease processes of HDP, i.e., increased creatinine and uric acid levels indicate renal dysfunction; diminished calcium levels reflect metabolic changes; and reduced placental growth factor (PIGF) indicates placental dysfunction (4).

Vitamin D is a secosteroid and plays pivotal role in bone and mineral metabolism. During pregnancy, vitamin D plays important role in implantation and placental function due to angiogenic, immunomodulatory, and anti-inflammatory effects (5). While still incompletely understood, the pathophysiology of HDP involves abnormal placentation and angiogenesis. Several studies have demonstrated an association between higher 25(OH)D levels and reduced risk of HDP especially in PE (6–11). Many studies reported that Indian subjects are more prone to vitamin D deficiency despite the availability of abundant sunshine throughout the year in many parts of India (12–17).

Hypertensive disorders in pregnancy are characterized as excessive maternal inflammatory responses to pregnancy. Inflammation involves a complex network of cytokines released from stressed cells and damaged tissues (18). Cytokines/chemokines are protein mediators that are known to be involved in many biological processes including cell growth, survival, inflammation, and differentiation (19). Limited data exist regarding cytokine/chemokine and metabolic hormone levels in subjects with hypertension disorders such as GH, PE, and EC. There is no study from Indian subjects to find the vitamin D association with circulatory cytokine/chemokine and metabolic hormone levels among healthy pregnancy (HP) associated with HDP. We inferred that vitamin D deficiency may be associated with systemic inflammation and could alter many cytokine/chemokine and metabolic hormones in hypertension disorders in pregnant women. To test our hypothesis, we have assessed serum cytokines/chemokines, metabolic hormones, and vitamin D metabolites in the HDP group (i.e., GH, PE, and EC) and compared those with the HP group and found the association of vitamin D metabolite levels with cytokine/chemokine and metabolic hormones.

## Materials and Methods

### Study Design

Cross-sectional study consists of pregnant women (HP, gestational hypertensive PE and EC subjects) in their third trimester, and inclusion criteria of subjects age was 18–45 years. Blood samples collected from women admitted with HP, GH, PE, and EC at the maternity ward at Mediciti Institute of Medical Sciences outpatient clinic, Hyderabad, India, were used for the study. GH was defined as development of hypertension (i.e., systolic BP ≥ 140 mmHg and diastolic BP ≥ 90 mmHg) for the first time after mid pregnancy (after 20 weeks). PE was defined as blood pressure ≥ 140/90 and proteinuria ≥ +1, measured at least twice 4–6 h apart after gestational week 20 (20). EC was defined as onset of convulsion in a woman with PE that cannot be attributed to other causes. None of the subjects had found complications with HELLP syndrome [A combination of the breakdown of red blood cells (hemolysis; the H in the acronym), elevated liver enzymes (EL), and low platelet count (LP) occurring in pregnancy]. HP group with normal BP, no proteinuria, and no systemic or endocrine disorder or absence of urinary tract infections were enrolled as control pregnant women. All healthy pregnant women will be in their third trimester (> 24weeks).

Women with previous PE pregnancies and who were on vitamin D supplements were not included. Women using acetylsalicylic acid or low-molecular weight heparin in the current pregnancy were also excluded from the study (4). Gestational age for both cases and controls were based on routine ultrasound examination between gestational week 17 and 20. Thirty serum samples from normal pregnant women individuals collected under informed consent were used as controls. Season of last menstrual period was defined as winter (December to February), spring (March to May), summer (June to August), and autumn (September to November).

The study was approved by the Mediciti Ethics Committee (Institutional). All women gave written informed consent for this study. The study did not include any vulnerable population. During the study, participants were interviewed about maternal age, chronic diseases, medication, ethnical origin, smoking status, method of conception, any previous pregnancies affected by PE or GH, and family history of PE.

### Blood and Urine Collection

#### Blood Sample Collection

After collection, the blood samples are allowed to stand for complete clot formation at room temperature and subsequently centrifuged for 10 min at approximately 1,500 × *g*. A total of 20 μL of each serum sample is used for creatinine and uric acid assay (within 3 h). Rest of the serum sample is appropriately labeled and stored at −80°C until batch analysis for vitamin D metabolites, serum cytokine/chemokine, and metabolic hormones measurement.

#### Urine Sample collection

A total of 10 mL of early morning midstream urine samples was collected into appropriately labeled sterilized containers and analyzed immediately for urine protein.

### Blood Pressure and Biochemical Parameter Measurement

Blood pressure was measured using oscillometric blood pressure monitor (model–omron HEM-780N3), calibrated prior to and once during the study period. Calibration of the instrument was checked periodically. Two measurements were taken after sitting comfortably on a chair for more than 5 min with left arm at the level of heart resting on a table. Serum creatinine levels were measured by modified kinetic Jaffe’s reaction in Dade Behring dimension Xpand plus system (creatinine flex reagent cartridge) according to the manufacturer’s instructions. Serum uric acid levels were measured by modified uricase method in Dade Behring dimension Xpand plus system (uric acid flex reagent cartridge) according to manufacturer’s instructions. Urinary protein levels have been measured by the pyrogallol red test. In brief, the concentration of total protein was determined by measuring the absorbance of colored solution at 600 nm by Systronics UV double beam spectrophotometry (21).

### Serum Cytokine/Chemokine and Metabolic Hormone Measurements

Serum levels of 27 cytokines, i.e., interleukin (IL)-1beta, IL-1 receptor antagonist (IL-1ra), IL-2, IL-4, IL-5, Il-6, IL-7, IL-8, IL-9, IL-10, IL-12, IL-13, IL-15, IL-17, eotaxin, basic FGF, G-CSF, GM-CSF, interferon (IFN)-gamma, IFN-gamma-inducible protein (IP)-10, monocyte chemotactic protein (MCP)-1, macrophage inflammatory protein (MIP)-1α, MIP-1β, platelet-derived growth factor (PDGF)-BB, rantes, tumor necrosis factor (TNF)-alpha, and vascular endothelial growth factor (VEGF), and 10 metabolic hormones, i.e., C-peptide, ghrelin, GIP, GLP-1, glucagon, insulin, leptin, plasminogen-activating factor (PAI)-1 (total), resistin, and visfatin were measured by using Bio-Plex Pro human cytokine Grp I panel 27-plex (Cat#M50-0KCAF0Y) and Bio-Plex Pro human diabetes panel 10-plex (Cat#171-A7001M), respectively. On the day of experiment, frozen serum samples were thawed, mixed by vortexing, and then centrifuged at 10,000 rpm for 5 min to isolate debris. All experiments were performed according to the manufacturer’s instructions using handheld magnetic separator bloc for 96-well flat bottom plates and analyzed using the Bio-Plex-200 system (Bio-Rad Corp.). All cytokine/chemokine and metabolic hormone standards were provided by the manufacturers. Acquisition gates were set at 8,000–15,000. Sample volume used for this analysis was 25 μl, and 50 events per bead were acquired. Mean fluoresce intensity was measured using Bio-Plex manager software version 5.0 (Bio-Rad) and compared to a standard curve to generate concentration values. Values below the range of the standard curve were set to the lower limit of detection. Three of the 27 cytokines analyzed (IL-2, GM-CSF, and IL-15) were excluded from further analyses, since approximately 95% sample concentration is below than the lowest standard or its maximum fluorescent intensity value near to background. The serum samples were measured in a single replicate, whereas cytokine standards and blank samples were measured in duplicate on each plate.

### UPLC/Atmospheric Pressure Chemical Ionization (APCI)/HRMS Method for Quantification of Vitamin D and Its Metabolite

Human serum (Biocell Laboratories, Inc., USA) was stripped and used as blank serum. Biocell serum was mixed with activated charcoal and agitated at room temperature overnight. The Biocell serum was then centrifuged at 9,000 × *g* for 20 min, and supernatant was removed and filtered. The filtered serum was again centrifuged at 6,000 × *g*, and the supernatant was separated. Stripped serum was injected into the UPLC/APCI/HRMS method to confirm untraceable level of vitamin D_3_ (VitD_3_) and vitamin D_2_ (VitD_2_) and its respective metabolites such as 25(OH)D_3_, 1,25(OH)_2_ D_3_, 25(OH)D_2_, and 1,25(OH)_2_ D_2_.

The stock solution of VitD_3_, VitD_2_, and its respective metabolites were prepared in ethanol. Serial dilution was done to prepare the primary aliquots for calibration curve samples ranging from 3 to 200 ng/mL for VitD_3_, 25(OH)D_3_, and VitD_2_ and 5–200 ng/mL for 1,25(OH)_2_ D_3_, 25(OH)D_2_, and 1,25(OH)_2_ D_2_. Similarly, low, middle, and high QC (LQC, MQC and HQC) samples at three different levels were prepared independently at concentrations of 3 ng/mL (LQC), 10 ng/mL (MQC), and 200 ng/mL (HQC) for VitD_3_, 25(OH)D_3_, and VitD_2_. Whereas, 5 ng/mL (LQC), 10 ng/mL (MQC), and 200 ng/mL (HQC) for 1,25(OH)_2_D_3_, 25(OH)D_2_, and 1,25(OH)_2_D_2_. All the stock solutions were stored at 0–4°C for further use. Stock solution of 1 mg/mL of dihydrotachysterol [Internal standard (IS)] was prepared in ethanol and diluted with methanol to prepare working solution containing a concentration of 50 ng/mL. To an aliquot of 100 μL of stripped serum, 10 μL of 50 ng/mL IS solution was added followed by 1 mL of hexane:heptane:acetone in the ratio of 45:40:15. This mixture was thoroughly mixed, shook, and centrifuged at 6,000 × *g* at 4°C. Evaporation of supernatant was done on ScanVac speed Vacuum Concentrator, and 100 μL of methanol was added and 10 μL aliquots of sample solution were injected into the UPLC/APCI/HRMS system for analysis.

Analysis was carried out on U-HPLC instrument (Thermo Scientific Accela, Germany) equipped with a quaternary pump, a degasser, a diode-array detector, an autosampler, and a column compartment. Mass spectrometric detection was carried out using an Orbitrap mass analyzer (Exactive Thermo Scientific, Germany) equipped with an APCI source. The data acquisition was under the control of Xcalibur software. The separation of vitamin D and its metabolites and IS from endogenous substances was achieved using Water’s X select CSH phenyl hexyl column (150 mm × 4.6 mm I.D.; particle size 3.5 μm) and mobile phase consisting of a mixture of ammonium formate 10 mM in methanol (A) and acetonitrile:acetone:IPA (5:4:1; B) in an gradient program mode. The gradient solvent program was set as follows: (T_min_/% proportion of solvent B): _0_/5, _0–5_/95, _5–6_/5, and _6–8_/5. The flow rate of the mobile phase was 1.00 mL/min, the column temperature 25°C, and the injection volume 10 μL. The typical operating source conditions for MS scan in positive ion APCI mode were optimized as follows sheath gas flow rate 65; Aux gas flow rate 20; discharge current 10.00 μA; capillary temperature 300°C; capillary voltage 50 V; tube lens voltage 85.0 V; skimmer voltage 18.00 V; and vaporizer temperature 380°C. For quantification, EICs of [M + H]^+^ at *m/z* 385.34649, [M + H-H_2_O]^+^ at *m/z* 383.33084, and [M + H-H_2_O]^+^ at *m/z* 399.32576 for VitD_3_, 25(OH)D_3_ and 1,25(OH)_2_D_3_, respectively with a 5 ppm range centered on the exact *m/z* value were generated. Similarly, EICs of [M + H-H_2_O]^+^ at *m/z* 379.33593, [M + H-H_2_O]^+^ at *m/z* 395.33084 and [M + H-H_2_O]^+^ at *m/z* 411.32576, and [M + H-H_2_O]^+^ at *m/z* 381.31519 for VitD_2_, 25(OH)D_2_ and 1,25(OH)_2_D_2_, and IS, respectively. The developed method was validated with respect to specificity, sensitivity, linearity, precision, accuracy, matrix effect, and stability. Details of the method were provided briefly in our previous publication (22).

### Statistical Analyses

Descriptive statistics of non-normally distributed data was reported as median (interquartile range), while normally distributed data were represented as mean ± SD and categorical variables as number (percentages). Data were tested for normality using the Kolmogorov–Smirnov test. Non-normal data were analyzed by Kruskal–Wallis test and Dunn’s test for pairwise comparisons. Spearman’s rank correlation coefficient was used to test for correlations between serum vitamin D metabolite levels and serum cytokine/chemokine and metabolic hormones with Bonferroni adjustment of multiple analysis. Univariate and multivariable logistic regression analyses were performed to identify covariates as potential confounders on the basis of their significance (*p* < 0.05). ORs with 95% CIs were used to report the results. Figures and tables were generated using GraphPad Prism v.5.0 and Matlab v.r2013b. SAS^®^ V.9.4 software was used for all other statistical analyses.

## Results

### Clinical Characteristics of the Study Population

Table 1 describes about the clinical characteristics of the study population. In the present study, 30 healthy pregnant women and 30 HDP (10 gestational hypertensive disorders, 10 PE, and 10 EC) subjects were enrolled for the study. Women participants’ age is ranging from 19 to 30 years. As expected, systolic and diastolic blood pressures were significantly higher in three different HDP as compared with the HP group (Table 1). In winter season, pregnant women with hypertension disorders were approximately 50% as compared to the other seasons. However, our data found 86.6% of healthy pregnant women are from winter season as compared to other seasons. Urinary microprotein levels significantly (*p* < 0.05) increased in PE and EC subjects as compared with the HP group and GH group. Serum uric acid and creatinine levels significantly (*p* < 0.05) increased in PE and EC subjects as compared with the HP group and significantly increased (*p* < 0.05) in EC as compared with the gestational hypertensive disorders group (Table 1).

**Table 1:** **Clinical characteristics of study population**.

Variables (characteristics at study visit)	Study groups			
	Healthy pregnancy (HP, *n* = 30)	Gestational hypertension (GH, *n* = 10)	Preeclampsia (*n* = 10)	Eclampsia (*n* = 10)
Age in years (mean ± SD)	21.80 ± 2.7	22.90 ± 3.7	23.00 ± 2.4	22.00 ± 2.7
Sys BP (mmHg) (mean ± SD)	116.3 ± 6.9	144.8 ± 1.3*	153.0 ± 7.9*	158.9 ± 3.4*
Dia BP (mmHg) (mean ± SD)	76.6 ± 5.0	95.10 ± 2.9*	100.5 ± 9.5*	103.6 ± 4.6*
Gestational age in weeks (mean ± SD)	36.67 ± 3.1	34.20 ± 4.1	33.0 ± 3.1	32.7 ± 4.9
Season of last menstrual cycle, *n* (%)				
Summer	0 (0)	0 (0)	1 (10)	2 (20)
Autumn	3 (10)	0 (0)	2 (20)	3 (30)
Spring	1 (3.3)	5 (50)	3 (30)	0 (0)
Winter	26 (86.6)	5 (50)	4 (40)	5 (50)
Proteinuria (dipstick)	n.a.	n.a.	2 (1–3)	2 (1–3)
Urinary microprotein (mg/dL)	7.4 (3.9–10.6)	2.2 (1.1–8.9)	131 (96.60–345.6)*^,^^†^	133.1 (101.0–309.9)*^,^^†^
Serum creatinine (mg/dL)	0.6 (06–0.7)	0.7 (0.6–0.8)	0.8 (0.7–0.8)*	0.8 (0.9–1.0)*^,^^†^
Serum uric acid levels (mg/dL)	3.8 (3.6–4.4)	4.6 (4.4–5.7)	6.3 (4.8–6.9)*	7.6 (6.8–8.3)*^,^^†^

*p < 0.05 compared to HP.

*^†^p < 0.05 compared to GH*.

*n.a., not applicable*.

*Mean ± SD for normally distributed variables and median with interquartile range for non-normally distributed data. Comparisons between outcome groups Kruskal–Wallis and Dunn’s test for continuous variables*.

### Distribution of 25(OH)D Levels in Normal Pregnant Women and Hypertensive Disorders in Pregnant Women

**Figure 1:**
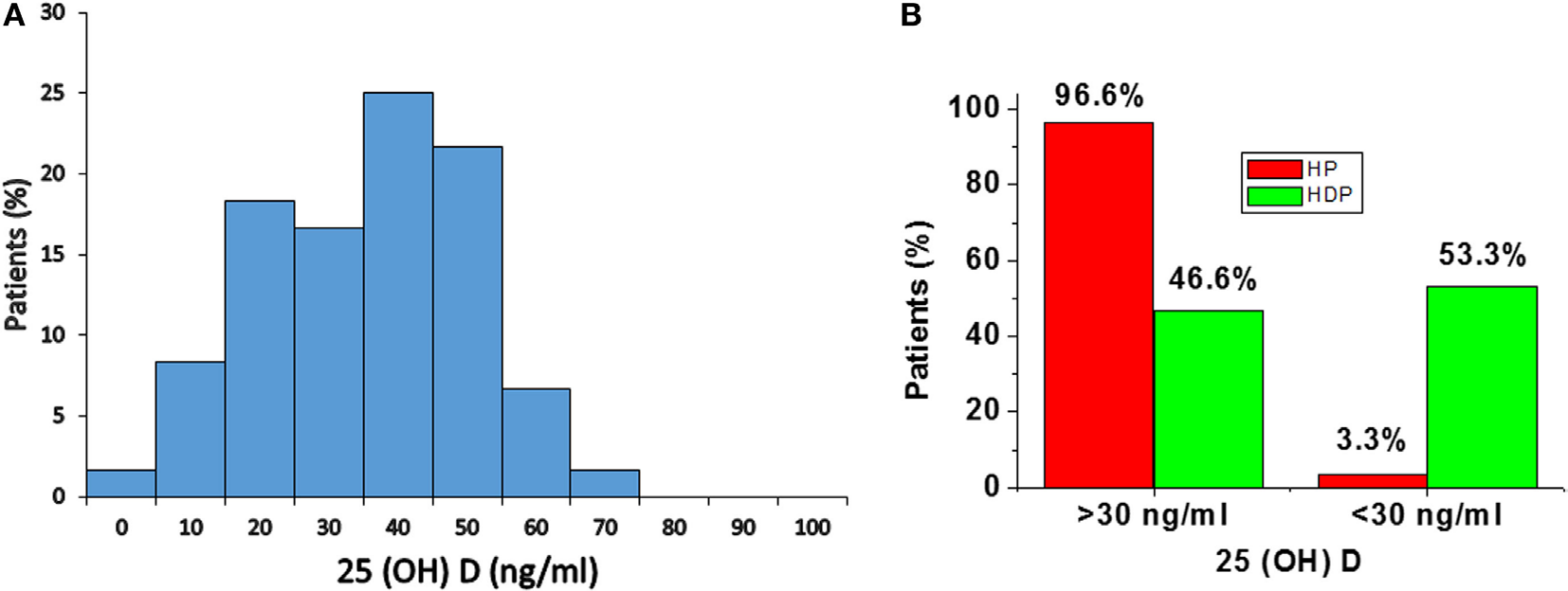
**(A)** Distribution of serum 25(OH)D concentrations in total subjects. **(B)** Distribution of 25(OH)D levels in healthy pregnancy (HP) and Hypertension disorders in pregnancy (HDP). Distribution of 25(OH)D levels according to serum concentrations, i.e., adequate (> 30 ng/mL) and inadequate (<30 ng/mL).

Distribution of total 25(OH)D levels in all subjects is mentioned in Figure 1. The median total 25(OH)D concentrations in the total subjects was 41.2 ng/mL (28.2–51.5) (Figure 1A). According to the previous literature, serum total 25(OH)D concentrations were well categorized as adequate (> 30 ng/mL) and inadequate <30 ng/mL (23).

In the present study, 96.6% of normal healthy pregnant women and 46.6% of hypertension disorders of pregnant women have adequate (> 30 ng/mL) level of 25(OH)D. A total of 3.3% of HP group and 53.3% of hypertensive disorders in pregnant women have inadequate (<30 ng/mL) level of 25(OH)D (Figure 1B).

### Vitamin D Metabolite Levels in Hypertensive Disorders in Pregnant Women

**Figure 2:**
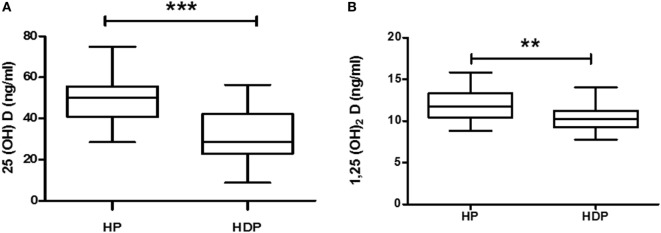
**Vitamin D metabolite (A) 25(OH)D and (B) 1,25(OH)_2_D levels in combined hypertension disorders in pregnancy (HDP) as compared to healthy pregnancy (HP)**. **p < 0.01, ***p < 0.001 vs HP. The Mann–Whitney *U* test and Student’s *t* test were used.

Total 25(OH)D is evaluated by sum of 25(OH)D + 25(OH)D_3_, and total 1,25(OH)_2_D is evaluated by sum of 1,25(OH)_2_D_2_ + 1,25(OH)_2_D_3_. Total 25(OH)D and total 1,25(OH)_2_D levels were decreased significantly (*p* < 0.05) in HDP group as compared with healthy pregnant group (Figures 2A,B).

Five vitamin D metabolites, i.e., VitD_3_, 25(OH)D_3_, 1, 25(OH)_2_D_3_, VitD_2_, and 1,25(OH)_2_D_2_ levels were significantly (*p* < 0.05) lower in the HDP group as compared with the HP group (Figures S1A–D,F in Supplementary Material). However, 25(OH)D_2_ did not show any alteration between the study groups (Figure S1E in Supplementary Material).

### Vitamin D Metabolite Levels in Normal Pregnant Women, GH, PE, and EC

**Figure 3:**
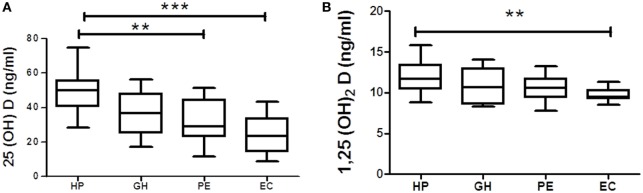
**Vitamin D metabolites (A) 25(OH)D and (B) 1,25(OH)2D in gestational hypertension (GH), preeclampsia (PE), and eclampsia (EC) as compared to the healthy pregnancy (HP) group**. **p < 0.01, ***p < 0.001 vs HP. For comparisons between outcome groups, the Kruskal–Wallis and Dunn’s tests were used.

Vitamin D metabolites from each hypertension disorder group were analyzed and compared with the HP group. Serum 25(OH)D levels were gradually decreased in three hypertensive disorders, i.e., GH, PE, and EC. However, significant (*p* < 0.05) reduction was observed only in PE and EC groups as compared with the HP group (Figure 3A). Serum 1,25(OH)_2_D levels were (*p* < 0.05) significantly decreased only in EC as compared with the HP group (Figure 3B).

Vitamin D_3_ levels significantly (*p* < 0.05) decreased in GH, PE, and EC subjects as compared with the HP group (Figure S2A in Supplementary Material). 25(OH)D_3_, VitD_2_, and 1,25(OH)_2_D_2_ levels significantly decreased in PE subjects as compared with the HP group (Figures S2B,D,F in Supplementary Material). VitD_2_ levels significantly (*p* < 0.05) decreased in EC subjects as compared with the gestational hypertensive subjects (Figure S2D in Supplementary Material). However, any alteration of 25(OH)D_2_ levels in hypertensive disorders in pregnant women was not found as compared with the HP group (Figure S2E in Supplementary Material).

### Serum Cytokine/Chemokine and Metabolic Hormone Profiles in HP, GH, PE, and EC Subjects

**Figure 4:**
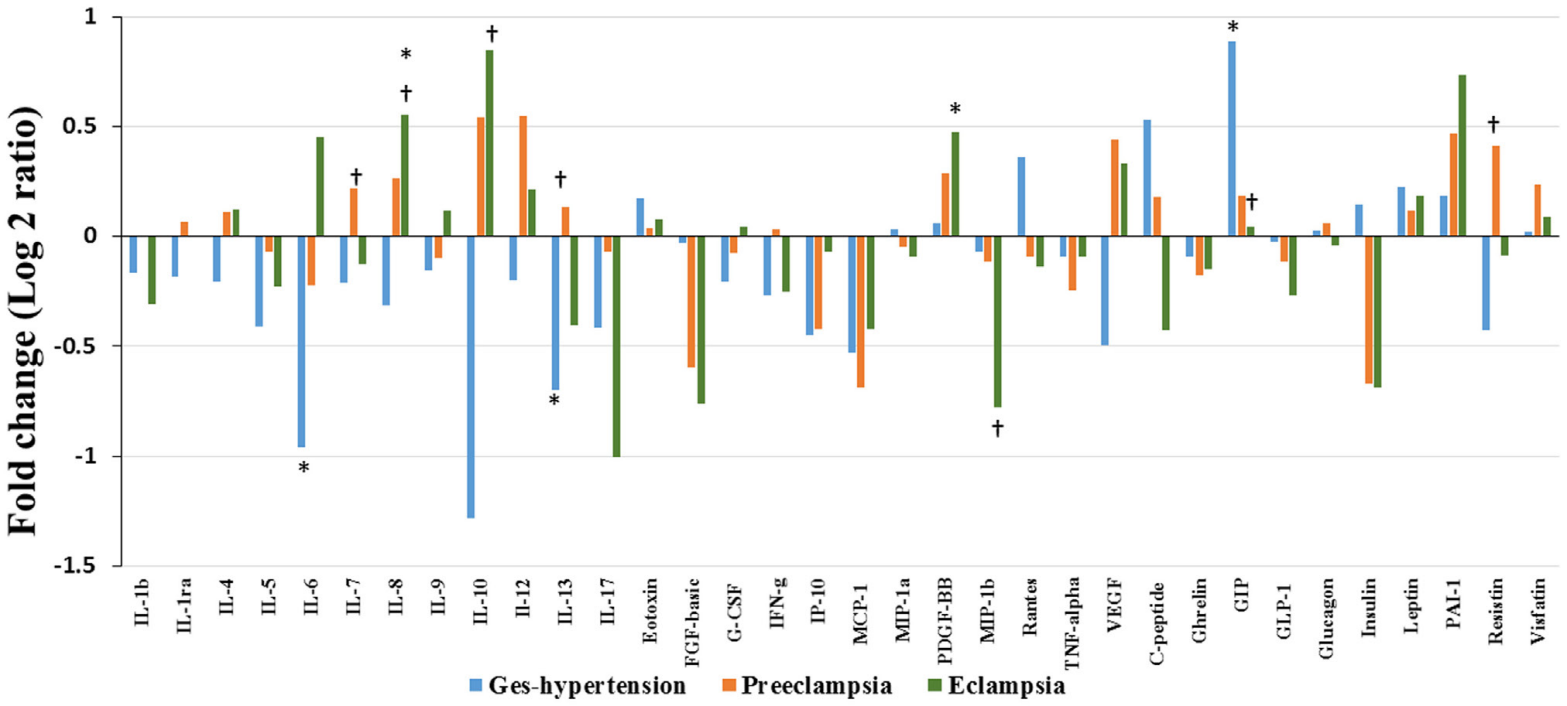
**Cytokine/chemokine and metabolic hormones expression: fold change expression of 34 analytes in gestational hypertension (GH), preeclampsia (PE), eclampsia (EC) compared to healthy pregnancy (HP) obtained by Bio-Plex assay**. Values more than 0 represent upregulation and less than 0 downregulation in three hypertension disorder groups, i.e., GH, PE, and EC. *<0.05 vs HP, ^†^0.05 vs GH.

Alteration of cytokines/chemokines and metabolic hormones has been compared among four groups. Serum levels of cytokine/chemokine and metabolic hormones are shown in Table 2. Fold change expressions of circulatory cytokine/chemokine and metabolic hormones are shown in Figure 4. Gestational hypertensive subjects have significantly lower serum IL-6, IL-13, and higher GIP levels as compared with the HP group. Surprisingly, there was no significant alteration of cytokine/chemokine and metabolic hormone levels in PE subjects as compared with the HP group. Serum IL-8 and PDGF-β levels significantly increased and MIP-1β levels decreased in EC subjects as compared with the HP. All serum parameters of PE and EC patients were compared with the GH group. IL-7, IL-13, and resistin levels significantly increased in PE subjects as compared with gestational hypertensive subjects. Serum rantes and GIP levels significantly decreased, and IL-8, IL-10 levels significantly increased in EC subjects as compared with the gestation hypertensive subjects. However, no significant changes in any serum parameters between PE and EC subjects (Table 2) were found. Significant alterations of cytokines/chemokines IL-1ra, IL-4, IL-5, IL-7, IL-9, IL-12, eotaxin, G-CSF, IFN-γ, IP-10, MIP-1α, TNF-α, VEGF, and metabolic hormones C-peptide, ghrelin, GIP, GLP-1, glucagon, leptin, and visfatin levels were not found in our study groups (Table 2). Significant alteration of plasma cytokine/chemokine and metabolic hormone levels among four groups, i.e., GH, PE, EC, and HP subjects is presented in Figures S3A–J in Supplementary Material.

**Table 2:** **Serum cytokine and metabolic hormone levels in study groups**.

Variables (pg/mL)	Healthy pregnancy (HP, *n* = 30)	Gestational Hypertension (GH, *n* = 10)	Preeclampsia (PE, *n* = 10)	Eclampsia (*n* = 10)
Interleukin (IL)-1β	2.04 (1.79–2.43)	1.82 (1.39–2.17)	2.04 (1.28–2.37)	1.65 (1.33–2.65)
IL-1 receptor antagonist	80.3 (70.6–110.9)	70.7 (59.8–79.4)	83.9 (65.2–101.8)	80.6 (49.7–113.1)
IL-4	1.96 (1.74–2.39)	1.70 (1.70–1.93)	2.12 (1.66–2.35)	2.13 (1.38–2.43)
IL-5	15.04 (12.6–16.8)	11.3 (8.55–15.9)	14.31 (9.73–17.19)	12.83 (9.16–17.85)
IL-6	4.72 (3.83–8.55)	2.425 (1.60–4.74)*	4.05 (2.21–7.12)	6.46 (3.72–9.18)
IL-7	26.87 (25.40–31.80)	23.15 (26.21–35.82)	31.31 (26.21–35.82)^†^	24.57 (19.95–4,032)
IL-8	12.76 (11.08–15.82)	10.28 (8.94–13.50)	15.34 (10.28–22.19)	18.71 (15.48–22.96)*^,^^†^
IL-9	24.08 (18.23–30.23)	21.63 (18.65–33.25)	22.47 (20.17–26.76)	26.06 (16.71–29.45)
IL-10	2.46 (1.44–4.27)	1.60 (0.91–2.42)	2.81 (1.99–8.01)	5.99 (2.39–10.05)^†^
IL-12	4.45 (3.19–9.09)	3.54 (2.68–6.95)	4.94 (2.76–11.07)	4.69 (0.88–11.68)
IL-13	41.86 (33.23–55.14)	25.27 (20.24–34.03)*	44.98 (34.03–55.10)^†^	30.92 (22.89–38.21)
IL-17	26.04 (16.42–38.13)	19.54 (14.92–25.13)	24.81 (20.75–38.23)	12.96 (6.025–29.63)
Eotaxin	50.38 (40.96–59.86)	56.89 (36.94–66.81)	51.78 (47.90–57.01)	53.15 (44.04–58.95)
FGF-basic	28.60 (15.35–42.35)	28.00 (21.06–38.56)	18.88 (8.93–32.44)	16.86 (15.35–29.77)
G-CSF	41.85 (37.29–48.28)	36.33 (31.74–38.13)	39.69 (34.64–52.30)	43.05 (32.10–50.28)
Interferon-gamma	145.0 (125.5–187.6)	120.5 (102.1–134.5)	148.3 (135.3–161.0)	121.9 (95.19–164.1)
IFN-gamma-inducible protein (IP)-10	1,247 (789.5–2,010)	914.5 (660.9–1,472)	932.4 (725.4–1,254)	1,187 (736.2–1,946)
Monocyte chemotactic protein -1	11.96 (7.2–15.75)	8.30 (7.42–10.22)	7.42 (6.15–8.30)	8.94 (3.2–12.32)
Macrophage inflammatory protein (MIP)-1α	2.21 (1.77–3.15)	2.26 (1.54–3.08)	2.14 (1.08–2.64)	2.07 (1.52–2.74)
Platelet-derived growth factor-BB	3,558 (2,757–4,597)	3,712 (3,117–4,385)	4,345 (3,204–5,884)	4,931 (4,493–6,098)*
MIP-1β	60.80 (48.85–78.97)	57.86 (45.99–75.21)	56.20 (39.09–70.96)	35.45 (30.31–53.31)*
Rantes	22,415 (17,813–26,419)	28,781 (20,391–29,826)	21,041 (20,394–24,885)	20,329 (15,136–21,372)^†^
Tumor necrosis factor-α	29.15 (23.63–34.76)	27.30 (21.82–33.82)	24.54 (20.46–25.46)	27.31 (17.34–34.30)
Vascular endothelial growth factor	3.285 (2.19–4.38)	2.33 (1.64–5.71)	4.46 (2.33–5.62)	4.14 (2.98–6.39)
C-peptide	2,157 (1,098–3,464)	3,109 (1,657–6,013)	2,438 (1,086–5,252)	1,601 (1,243–2,938)
Ghrelin	574.3 (448.7–777.6)	539.3 (400.2–657.4)	507.3 (432.1–668.4)	517.0 (450.9–656.2)
GIP	362.0 (243.5– 498.4)	670.3 (571.3–1,116)*	411.1 (306.5–875.3)	373.0 (203.6–425.0)^†^
GLP-1	296.3 (267.8–326.1)	290.7 (263.7–320.4)	273.3 (238.5–284.9)	246.3 (233.9–331.3)
Glucagon	298.3 (279.5–329.4)	303.8 (271.7–318.9)	310.6 (280.7–330.0)	289.4 (252.5–308.0)
Insulin	1,155 (646.5–2,305)	1,275 (788.7–4,890)	725.6 (543.9–1,932)	718.1 (428.3–1,420)
Leptin	11,068 (4,872–19,974)	12,913 (8,028–21,469)	12,015 (8,297–29,339)	12,588 (3,447–46,832)
Plasminogen-activating factor-1	1,02,521 (75,285–1,64,409)	116,552 (98,060–306,583)	1,41,578 (84,497–2,33,429)	170,343 (118,350–240,203)
Resistin	9,584 (6,531–12,557)	7,119 (5,474–9,264)	12,765 (10,003–18,040)^†^	9,025 (6,421–12,162)
Visfatin	1,469 (1,186–2,314)	1,492 (1,224–2,449)	1,732 (1,488–2,389)	1,561 (1,163–1,937)

*Continuous variables are reported as median (25th–75th percentile) as not found normally distributed. Comparisons between outcome groups by the Kruskal–Wallis and Dunn’s tests*.

*p < 0.05 compared to HP.

*^†^p < 0.05 compared to GH*.

### Serum Cytokine/Chemokine and Metabolic Hormone Profiles in Combined Hypertensive Disorders of Pregnancy As Compared with the HP Group

In the present study, serum cytokine/chemokine and metabolic hormone levels in the combined hypertensive disorders group were compared with the HP group. Cytokine/chemokine IL-1ra, IL-6, IL-13, IFN-γ, MCP-1, MIP-1β levels and metabolic hormone such as GLP-1 levels significantly (*p* < 0.05) decreased in pregnant women with hypertensive disorders as compared with the HP group (Table 3). Serum PAI-1 and GIP levels were increased in the hypertension disorders group as compared with the HP group (Table 3).

**Table 3:** **Serum cytokine/chemokine and metabolic hormone levels in healthy pregnancy (HP) and hypertension disorder in pregnancy**.

Variables (characteristics at study visit) (pg/mL)	HP (*n* = 30)	Hypertension disorders in pregnancy (*n* = 30)
Interleukin (IL)-1β	2.04 (1.79–2.43)	1.87 (1.39–2.26)
IL-1 receptor antagonist	90.58 (71.7–110.9)	70.07 (59.8–96.73)^a^
IL-4	1.96 (1.74–2.39)	1.83 (1.43–2.32)
IL-5	15.04 (12.6–16.8)	12.46 (8.57–16.4)
IL-6	4.72 (3.83–8.6)	4.16 (2.16–7.10)^a^
IL-7	26.87 (25.40–31.80)	25.23 (21.44–34.02)
IL-8	12.76 (11.08–15.82)	14.48 (10.28–20.31)
IL-10	2.53 (1.54–4.42)	2.53 (1.99–7.21)
Il-12	4.45 (3.195–9.09)	3.72 (2.68–8.16)
IL-9	24.08 (18.23–30.23)	22.08 (19.48–27.39)
IL-13	41.86 (33.23–55.14)	31.19 (23.94–40.08)^a^
IL-17	26.04 (16.42–38.13)	22.37 (14.92–29.42)
Eotaxin	50.38 (40.96–59.86)	54.12 (43.19–58.72)
FGF-basic	28.60 (15.35–42.35)	22.73 (15.75–33.17)
G-CSF	41.85 (37.29–48.28)	39.69 (32.95–44.00)
Interferon (IFN)-gamma	145.0 (125.5–187.6)	135.3 (102.1–157.8)^a^
IFN-gamma-inducible protein (IP)-10	1,247 (789.5–2,010)	978 (699.6–1,428)
Monocyte chemotactic protein-1	11.96 (7.2–15.75)	7.16 (5.93–10.75)^a^
Macrophage inflammatory protein (MIP)-1α	2.21 (1.77–3.15)	2.07 (1.52–2.74)
Platelet-derived growth factor-BB	3,558 (2,757–4,597)	4,295 (3,242–5,326)
MIP-1β	60.80 (48.85–78.97)	54.06 (33.49–60.07)^a^
Rantes	22,415 (17,813–26,419)	21,170 (20,197–27,703)
Tumor necrosis factor-alpha	29.15 (23.63–34.76)	25.46 (20.01–30.08)
Vascular endothelial growth factor	3.285 (2.19–4.38)	3.83 (2.19––5.79)
C-Peptide	2,157 (1,098–3,464)	1,897 (1,447–4,650)
Ghrelin	574.3 (448.7–777.6)	517.8 (446.7–606.7)
GIP	362.0 (243.5– 498.4)	443.4 (317.3–830.3)^a^
GLP-1	296.3 (267.8–326.1)	271.1 (242.8–316.7)^a^
Glucagon	298.3 (279.5–329.4)	300.7 (271.7–318.9)
Insulin	1,155 (646.5–2,305)	913.3 (552.2–2,248)
Leptin	11,068 (4,872–19,974)	12,913 (7,738–28,037)
Plasminogen-activating factor-1	102,521 (75,285–1,64,409)	1,47,652 (1,01,855–2,40,203)^a^
Resistin	9,584 (6,531–12,557)	9,318 (6,095–12,101)
Visfatin	1,469 (1,186–2,314)	1,612 (1,254–2,070)

*Continuous variables are reported as median (25th–75th percentile). Mann–Whitney *U* test was used to determine statistical significance*.

*^a^Significant at 5% level*.

### Correlations between Vitamin D Metabolites and Cytokine/Chemokine, Metabolic Hormones, Clinical Characteristics, and Laboratory Parameters

Correlations between vitamin D metabolites with various clinical parameters, cytokines/chemokines and metabolic hormones are presented in Table 4. There were significant negative correlation between 25(OH)D and parameters like SBP (*r* = −0.625, *p* < 0.0001) and DBP (*r* = −0.632, *p* < 0.0001) in overall subjects (Table 4; Figure S4 in Supplementary Material), similarly, serum creatinine (*r* = −0.470, *p* < 0.0001) and serum urea levels (*r* = −0.528, *p* < 0.0001) were also negatively correlated with 25(OH)D. Also, significant negative correlations were observed between 1,25(OH)_2_D and parameters like SBP (*r* = −0.449, *p* = 0.0003) and DBP (*r* = −0.425, *p* = 0.0007) in overall subjects (Table 4). Significance was reported with Bonferroni adjustment taking into account for multiple testing.

**Table 4:** **Correlations between vitamin D metabolites and circulatory markers of all study subjects**.

Variables	Correlation coefficient	*p*-Value
25(OH)D vs SBP	−**0.625**	**<0.0001**
25(OH)D vs DBP	−**0.632**	**<0.0001**
25(OH)D vs UMP	−0.348	0.0064
25(OH)D vs serum creatinine	−**0.470**	**<0.0001**
25(OH)D vs serum urea	−**0.528**	**<0.0001**
25(OH)D vs monocyte chemotactic protein-1	0.274	0.0405
25(OH)D vs macrophage inflammatory protein (MIP)-1α	0.350	0.0076
25(OH)D vs MIP-1β	0.273	0.0343
25(OH)D vs tumor necrosis factor-alpha	0.342	0.0097

1,25(OH)_2_D vs SBP	−**0.449**	**0.0003**
1,25(OH)_2_D vs DBP	−**0.425**	**0.0007**
1,25(OH)_2_D vs serum creatinine	−0.354	0.0054
1,25(OH)_2_D vs serum urea	−0.272	0.0352
1,25(OH)_2_D vs interleukin (IL)-9	0.257	0.0470
1,25(OH)_2_D vs IL-17	0.259	0.0450
1,25(OH)_2_D vs interferon-γ	0.267	0.0386
1,25(OH)_2_D vs MIP-1β	0.331	0.0096
1,25(OH)_2_D vs ghrelin	−0.264	0.0409

*Bold letters represents statistical significance taking Bonferroni adjustment into account*.

When significance was considered as *p* < 0.05, total 25(OH)D shows positive correlation with MCP-1 (*r* = 0.274, *p* = 0.0405), MIP-1α (*r* = 0.350, *p* = 0.0076), MIP-1β (*r* = 0.273, *p* = 0.0343), and TNF-α (*r* = 0.342, *p* = 0.0097) levels. Significant (*p* < 0.05) negative correlations were observed between 1,25(OH)_2_D with serum creatinine (*r* = −0.354, *p* = 0.0054), serum urea (*r* = −0.272, *p* = 0.0352), and ghrelin (*r* = −0.264, *p* = 0.0409) levels, while positive correlations were observed between 1,25(OH)_2_D and parameters such as IL-9 (*r* = 0.257, *p* = 0.0470), IL-17 (*r* = 0.259, *p* = 0.0450), INF-γ (*r* = 0.267, *p* = 0.0386), and MIP-1β (*r* = 0.331, *p* = 0.0409) levels (Table 4). However, correlation between vitamin D metabolites and inflammatory markers was not found significant taking Bonferroni adjustment into account for multiple testing.

### Groupwise Correlations between Blood Pressure Parameters and Circulatory Markers

Correlation analysis, between 25(OH)D with various blood pressure parameters in hypertension disorder groups, reveals that only 25(OH)D is negatively associated with SBP (*r* = −0384, *p* = 0.0362) and not with DBP (Figure S5 in Supplementary Material). However, there is no association found between 25(OH)D and SBP, DBP in healthy pregnant group (Figure S6 in Supplementary Material).

Correlation analysis between blood pressure parameters and circulatory markers in the HP group shows that only diastolic blood pressure is negatively correlated with INF-γ (*r* = −0.540, *p* = 0.0020) with Bonferroni adjustment for multiple testing. Similarly, in the hypertensive group, systolic blood pressure was positively associated with urinary microprotein (*r* = 0.712, *p* < 0001), serum creatinine (*r* = 0.667, *p* < 0.0001), and serum urea (*r* = 0.664, *p* < 0.0001). The diastolic blood pressure of the disease group positively correlated with serum creatinine (*r* = 0.607, *p* < 0.0004) and serum urea (*r* = 0.548, *p* < 0.0017) levels (Table 5).

**Table 5:** **Groupwise correlations between blood pressure parameters and circulatory markers of all study subjects**.

	Correlation coefficient	*p*-Value
**Healthy pregnancy variables**		
SBP vs serum creatinine	0.487	0.0063
SBP vs interferon-gamma (INF-γ)	−0.445	0.0135
SBP vs vascular endothelial growth factor (VEGF)	−0.424	0.0243

DBP vs interleukin 1 receptor antagonist	−0.369	0.0483
DBP vs interleukin (IL)-5	−0.388	0.0374
DBP vs IFN-γ	−**0.540**	**0.0020**
DBP vs tumor necrosis factor-alpha	−0.398	0.0393
DBP vs VEGF	−0.421	0.0256

**Hypertension disorders in pregnancy variables**		
SBP vs UMP	**0.712**	<**0.0001**
SBP vs serum creatinine	**0.667**	<**0.0001**
SBP vs serum urea	**0.664**	<**0.0001**
SBP vs IL-6	0.447	0.0133
SBP vs IL-10	0.556	0.0048
SBP vs 25(OH)D	−0.384	0.0357

DBP vs UMP	0.515	0.0035
DBP vs serum creatinine	**0.607**	**0.0004**
DBP vs serum urea	**0.548**	**0.0017**
DBP vs IL-10	0.434	0.0341
DBP vs FGF-basic	−0.430	0.0358
DBP vs GM-CSF	0.474	0.0221

*Bold letters represents statistical significance taking Bonferroni adjustment into account*.

However, considering the significance with (*p* < 0.05), SBP is positively correlated with serum creatinine (*r* = 0.487, *p* < 0.0063) and negatively correlated with INF-γ (*r* = −0.445, *p* = 0.0135) and VEGF (*r* = −0.424, *p* < 0.0243) in the HP group. DBP correlated with IL-1ra (*r* =−0.369, *p* < 0.0483), IL-5 (*r* = −388, *p* < 0.0374), TNF-α (*r* = −0398, *p* < 0.0393), and VEGF (*r* = −0.421, *p* < 0.0256) in the HP group. In the hypertension disorders group, significantly SBP is negatively correlated with IL-6 (*r* = 0.447, *p* < 0.0133) and IL-10 (0.556, *p* < 0.0048). DBP is positively correlated with UMP (*r* = 0.515, *p* < 0.0035), IL-10 (*r* = 0.434, *p* < 0.0341), and GM-CSF (*r* = 0.474, *p* < 0.0221) and negatively correlated with FGF-basic (*r* = −0.430, *p* < 0.0358) (Table 5).

### Fold Change Alteration of Cytokine/Chemokine and Metabolic Hormones Pattern among Three HDP Groups

**Figure 5:**
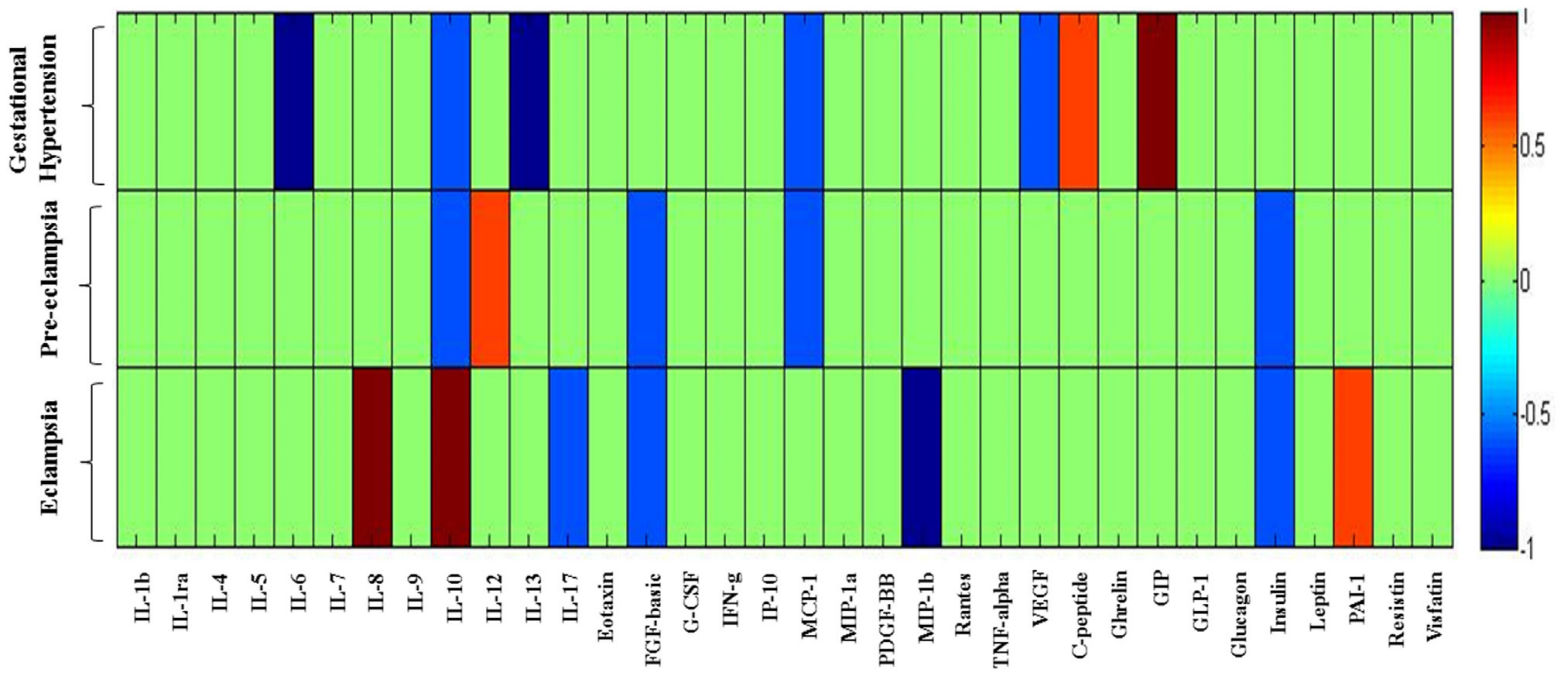
**Heat map provides a graphical representation of the secretary cytokine/chemokine and metabolic hormones pattern showing a fold change cutoff of ≥1.4 in three hypertension disorders (gestational hypertension, preeclampsia, and eclampsia) in pregnant women as compared to the healthy pregnancy group**. Hypertension disorder groups represented in *y*-axis and cytokine/chemokine and metabolic hormones on *x*-axis. 1 represents upregulated parameters and −1 represents downregulated. Changes in markers are indicated using the following colors: dark blue, significantly downregulated; light blue, non-significantly downregulated; dark red, significantly upregulated; light red, non-significantly upregulated.

In the previous section, it is shown that the several significant markers perturbed in different hypertension disorder groups as compared with the HP group. To identify more biologically relevant parameters perturbed in disease condition, two criterion were used (i) alteration should be statistically significant (*p* < 0.05) and (ii) fold change should be ≥ 1.4. Both criteria were used to eliminate background noise to identify markers for specific disease condition. When we consider only fold change ≥ 1.4 as criteria, IL-6, IL-10, IL-13, MCP-1, VEGF, C-peptide, and GIP levels were altered in GH as compared with the HP group (Figure 5). However, very few markers such as IL-6, IL-13, and GIP levels were matched with the abovementioned two criteria. Similarly, when only fold change cutoff was considered, IL-10, IL-12, FGF-basic, MCP-1, and insulin were altered in PE subjects as compared with the HP group (Figure 5). However, no markers were identified after consideration of both the cutoffs. Considering only fold change cutoff, IL-8, IL-10, IL-17, FGF-basic, MIP-1β, insulin, and PAI-1 markers were identified in EC subjects as compared with the HP group (Figure 5). However, after applying both fold change and significance cutoff, only IL-8, IL-10, and MIP-1β were altered. Our results indicate that fold change cutoff ≥ 1.4 is proved to be a better eliminator of background noise as there were fewer markers left after making a fold change cutoff of ≥ 1.4 as compared to using significance cutoffs (Table 1). As the fold change level increases to that of ≥ 2, the number of markers significantly decreases. Parameters that change in the significance level along with the fold change cutoffs provide list of biomarkers that imply inflammatory signaling pathways and functional involvement of hypertensive disorders of pregnant women.

### Logistic Regression Analysis

Multivariable–multivariate logistic regression analyses were performed to test the association of the vitamin D metabolites and cytokine/chemokine and metabolic hormones with the presence of HDP. Table 6 shows various models using univariate and multivariable–multivariate logistic regression analyses. The unadjusted univariate analysis shows that INF-γ [OR 0.98 (CI 0.96–0.99) *p* = 0.0344], MCP-1 [OR 0.87 (CI 0.77–0.98) *p* = 0.0237], 25(OH)D [OR 0.88 (CI 0.88–0.94) *p* = 0.0001], and 1,25(OH)_2_D [OR 0.62 (CI 0.44–0.86) *p* = 0.0044] were found to be significant. However, univariate adjusted analysis reveals that IL-17 [OR 0.93 (0.88–0.99) *p* = 0.0229], MCP-1 [OR 0.76 (0.628–0.927) *p* = 0.0066], 25(OH)D [OR 0.83 (0.80–0.95) *p* = 0.0016], and 1,25(OH)_2_D [OR 0.67 (0.46–0.98) *p* = 0.0370] were found to be significant when adjusted with gestational age and season of last menopausal period. Two multivariable-adjusted models are shown in Table 6 adjusted with gestational age and season of last menopausal period. In the model 1, eotaxin [OR 1.07 (1.01–1.13) *p* = 0.0092], MCP-1 [OR 0.681 (0.479–0.966), *p* = 0.0315], and 25(OH)D [OR 0.857 (0.758–0.969) *p* = 0.0141] were predictors of HDP, whereas model 2 confirms that eotaxin [OR 1.06 (1.01–1.11) *p* = 0.0154] and MCP-1 [OR 0.703 (0.546–0.904), *p* = 0.0060] are predictors of HDP with 1, 25(OH)_2_D [OR 0.457 (0.239–0.876) *p* = 0.0023].

**Table 6:** **Logistic regression analysis of HP vs HDP subjects**.

	Univariate analysis				Multivariable analysis			
	Unadjusted		Adjusted		Adjusted			
					Model 1		Model 2	
			
Factor	OR (95% CI)	*p*-Value	OR (95% CI)	*p*-value	OR (95% CI)	*p*-Value	OR (95% CI)	*p*-Value
IL-1 receptor antagonist (pg/mL)	0.98 (0.96–1.00)	0.0785	0.986 (0.966–1.006)	0.1656				
Interleukin (IL)-13 (pg/mL)	0.97 (0.94–1.00)	0.0776	0.973 (0.938–1.009)	0.1464				
IL-17 (pg/mL)	0.96 (0.92–1.00)	0.0600	**0.936 (0.884–0.991)**	**0.0229**				
Eotaxin (pg/mL)	1.02 (0.99–1.04)	0.0706	1.036 (0.999–1.074)	0.0597	**1.072 (1.017–1.130)**	**0.0092**	**1.060 (1.011–1.112)**	**0.0154**
Interferon-gamma (pg/mL)	**0.98 (0.96–0.99)**	**0.0344**	0.989 (0.971–1.007)	0.2253				
Monocyte chemotactic protein-1 (pg/mL)	**0.87 (0.77–0.98)**	**0.0237**	**0.763 (0.628–0.927)**	**0.0066**	**0.681 (0.479–0.966)**	**0.0315**	**0.703 (0.546–0.904)**	**0.0060**
Tumor necrosis factor-alpha (pg/mL)	0.93 (0.87–1.00)	0.0723	0.948 (0.869–1.034)	0.2273				
GIP (pg/mL)	1.00 (1.000–1.003)	0.0509	1.001 (0.999–1.00)	0.2767				
25(OH)D (ng/mL)	**0.886 (0.883–0.942)**	**0.0001**	**0.837 (0.806–0.951)**	**0.0016**	**0.857 (0.758–0.969)**	**0.0141**		
1,25(OH)_2_D (ng/mL)	**0.62 (0.446–0.862)**	**0.0044**	**0.674 (0.463–0.980)**	**0.0370**			**0.457 (0.239–0.876)**	**0.0023**

*All adjusted models (including univariate and multivariable) were adjusted with gestational age and last menstrual period.Bold letters represents significance p < 0.05*.

## Discussion

Hypertensive disorders of pregnancy are more common and morbid complications of pregnancy. GH is the early stage of hypertension, and PE is a combination of PE with proteinuria. EC is the advance and severe phase of PE. The mechanisms that are underlying the disease causation and progression are largely unknown. There are currently no early diagnostic tests that can identify women at increased risk. Many studies previously reported that low levels of vitamin D have been found in both males and females. However, the prevalence is higher among females and this may increase the risk of different pregnant disorders including PE (7). The link between lower vitamin D levels and hypertension has already been reported. Lower vitamin D was associated with hypertension in different population (24–26). Thus, there is a high possibility that lower vitamin D levels may also increase hypertension in pregnant women. In the present study, we measured six vitamin D metabolites in the HP group and three hypertensive disorders in pregnant women, i.e., GH, PE, and EC. Significant decrease in all vitamin D metabolite levels were observed in pregnant women with hypertensive disorders except 25(OH)D_2_. Total 25(OH)D decreased significantly in PE and EC as compared with the HP group. However, the active vitamin D metabolites, i.e., 1,25(OH)_2_D levels, significantly decreased only in EC subjects. Overall, our data confirming that vitamin D deficiency may contribute to the development of hypertensive disorders in pregnant women. Our results were consistent with previous results, which reported that VitD_3_ levels significantly decreased in PE and EC subjects (27), and lower maternal vitamin D deficiency is the independent risk factor for the PE (7). It could also be possible that decreased vitamin D levels might be due to hypertension-produced stress on the kidney. Impaired renal function may cause the decrease in the levels of vitamin D in blood. However, in our study we have not included subjects with kidney problem and none of the pregnant women have creatinine levels > 1.5 mg/dL. Thus, the possibility to find lower vitamin D metabolite levels in hypertensive disorder patients due to the stress or mild injury of kidney is much less.

Vitamin D plays a crucial role in controlling anti-inflammatory and pro-inflammatory cytokines balance (28). Previous literature reported that adequate vitamin D level is essential for the prevention of inflammatory diseases by modulating many genetic, immune, and inflammatory responses through vitamin D receptors (VDRs) (29–31). VDR is widely presented in all tissues in the body including macrophages, neutrophils, dendritic cells, and T-lymphocytes. The availability of VDR in the immune cells confirms that vitamin D plays a pivotal role in functioning in the immunity system including regulation of cytokine environment (32). Many mechanisms were involved in the prevention or delaying the progression of PE. One of the important mechanisms that proposed is related to a defective control of effector T cells by regulatory T cells, which may cause poor placental invasion and lead to release placental-derived vasoconstrictor factors. All these factors promote and enhance maternal hypertension and proteinuria (33, 34). As vitamin D plays an important role in maintaining and restoring immune homeostasis and tolerance, the availability and effectiveness of calcitriol may directly regulate the immune cell function in HDP (33, 35). Another hypothesis is that lower vitamin D provides hypertensive effect through activation of renin–angiotensin–aldosterone system (RAAS). There are several human studies reported that increased circulatory levels of renin and angiotensin II associated with hypertension (36, 37). The molecular mechanism of vitamin D in the RAAS inhibition is through the suppression of renin expression by liganded VDR through binding to the transcription factor cAMP-response element-binding protein (38). Therefore, RAAS activation may cause abnormal endothelial function and is responsible for hypertensive disorder in pregnancy (39). Similarly, increased inflammatory cytokine levels may trigger endothelial dysfunction. 1,25(OH)_2_D_3_ acts directly with VDR on the T lymphocyte to inhibit its proliferation (40, 41). Recently, researchers also reported that 1,25(OH)_2_D_3_ inhibits production of pro-inflammatory cytokines such as IFN-γ, IL-17, and IL-21 in CD4^+^CD25^−^ T lymphocytes (42). Thus, lower vitamin D levels observed in the HDP group independently regulate hypertension in pregnancy and inflammation. However, we cannot comment on whether inflammation caused by lower vitamin D levels influences hypertension.

Cytokines/chemokines are cell signaling molecules play important role in modulating all immune responses. In general, cytokine/chemokine present in circulation picomole concentration but may increase to more than 1,000-fold during inflammation condition (43). A group of cytokines work as pro-inflammatory while others work as anti-inflammatory modulators. GH, PE, and EC are excessive maternal inflammatory responses to pregnancy (4). In the present study, 27 cytokines/chemokines and 10 metabolic hormones were measured to know the alteration in HDP. IL-6 and IL-13 levels significantly decreased in combined hypertension disorders when compared with normal pregnant women. In individual hypertension disorders, significantly lower levels of IL-6 and IL-13 were observed only in the GH group but not in PE and EC groups when compared with normal pregnant women. Unlike our study, Tangeras et al. did not find IL-6 alteration in GH and PE patients as compared with normal pregnant women (4). It could be possible that decrease in the IL-6 levels in GH is an immediate acute phase response to the early-stage hypertension, and these levels were further improved gradually in the PE and EC groups. IL-6 may act as both pro-inflammatory and anti-inflammatory cytokines (44) and control angiogenic balance and endothelial regulation in hypertensive pregnant women (45). Similarly, decreased IL-13 levels in the GH group and then increased levels in PE as compared to GH indicate compensatory responses to modulate the inflammation in HDP (46). Pro-inflammatory cytokine IL-8 levels increased in PE and EC subjects as compared to the normal pregnant women. This is consistent with the previous data reported by Tosun et al., where IL-8 level was higher in severe PE as compared with control and mild PE (47). These findings suggest that IL-8 may increase with severity of HDP. Similarly, IL-10 levels gradually increased from mild to severe hypertension disorder groups. However, significant changes were observed in the EC group as compared to the GH. Previous studies reported contradictory data in the circulatory IL-10 levels. While some reported higher levels of IL-10 in PE (48), whereas others reported lower (49–51). This variation in different studies might be due to sampling in different stages of pregnancy. IL-10 plays an important role in reduction of excessive inflammation by inhibiting IL-1, IL-6, IL-12, TNF-α, and chemokines. IL-10 works as a protective agent during inflammation (52). IL-10 regulates maternal immunity through maintenance of uterine NK cell and reduces their cytotoxic functions in response to pro-inflammatory challenges during pregnancy (53–55). Furthermore, increased IL-10 production in decidual Treg cells may inhibit immune stimulation of T cells (56). In the present study, increased IL-10 levels in hypertension disorders, i.e., EC in pregnant women might be due to protective responses against inflammation.

Increased PDGF-BB levels were observed in EC subjects as compared with the HP group. Increased PDGF-BB levels play an important role in the vascular structural changes associated with hypertension (57). Our data showed that PDGF-BB levels increased gradually as the severity of the hypertension disorders increases.

When we considered all significant (*p* < 0.05) altered parameters with a ≥ 1.4 fold change, inflammatory markers such as IL-6, IL-13, MCP-1, and metabolic hormones such as GIP and C-peptide play important role in GH. However, we could not detect any markers that altered in PE. In case of EC, IL-8 and MIP-1β play major role for the pathogenesis. This clearly indicates that inflammation plays a crucial role in HDP and these alterations were more in early hypertension disorders and severity of EC conditions. In the present study, correlation of vitamin D metabolites with other parameters such as blood pressure, cytokine/chemokines, and metabolic hormones was calculated. Upon considering significance (*p* < 0.002) with Bonferroni adjustment, negative correlation was observed between vitamin D metabolites [total 25(OH)D and 1,25(OH)_2_D] and clinical characteristics such as systolic blood pressure and diastolic blood pressure. However, only 25(OH)D is negatively correlated with serum creatinine and serum urea levels. When significance (*p* < 0.05) was considered, 25(OH)D is negatively correlated with UMP and positively correlated with inflammatory markers MCP-1, MIP-1α, MIP-1β, and TNF-alpha. Similarly, 1,25(OH)_2_D is negatively correlated with creatinine, urea, and ghrelin and positively correlated with IL-9, IL-17 IFN-γ, and MIP-1β. These data confirm that correlation of lower vitamin D and its metabolite levels with clinical characteristics and laboratory parameters of GH, PE, and EC in Indian pregnant women. Data from the present study confirm that there is a strong association between cytokines and vitamin D metabolites. Recently, Zerofsky et al. reported that vitamin D supplementation with 2,000 IU/day associated with increased regulatory T cell immunity (58), which may prevent the adverse outcomes caused by excess inflammation.

After that it tried to find if there is any correlation between groupwise correlations of blood pressure vs circulatory markers. In the HP group, DBP negatively correlated with IFN-γ. However, in the case of the HDP group, both SBP and DBP positively correlated with serum creatinine and serum urea, and hypertensive disorders in pregnant women DBP positively correlated with urinary microprotein. These findings suggest that decreased vitamin D levels may increase blood pressure in pregnant women. Our results are consistent with the previous literature (7).

Finally, logistic regression analysis was performed to get causal relationship between hypertension disorders and HP. The analysis revealed that eotaxin, MCP-1, and vitamin D metabolites 25(OH)D and 1,25(OH)_2_D were common predictors of hypertensive disorders in pregnant women as compared with the HP group. Other cytokines/chemokines IL-1ra, IL-13, IL-17, INF-γ, TNF-α, and GIP were near to the significance levels. Future studies with large number of subjects will find the association of these abovementioned markers with vitamin D level in pregnant women.

Although some good correlations have been found between vitamin D metabolites and cytokine levels, the present study had some limitations. It was a single-center one-point study among South Indian subjects. Thus, the results may not be generalizable to other population. Another limitation of this study is the relatively small number of cases, but these numbers were found to be comparable to previous case–control studies (59, 60). The issue of whether GH and PE are separate disorders with the common clinical sign of hypertension, or part of a spectrum, has not been settled. Our findings strongly suggest that these hypertensive pregnancy disorders should be addressed separately with large number of subjects in future studies. Most importantly, as an observational study, this study is more hypothesis generating and does not address causality. Another limitation of this study is that we have measured freely available circulatory cytokine/chemokine levels and not measured those from micro-/macrovesicles and exosomes, which may excrete during stress conditions in pregnancy. Finally, we recognize the continued uncertainty as the optimal method of measuring 25(OH)D levels (61, 62); however, we used the established gold standard UPLC/APCI/HRMS method to measure six vitamin D metabolites and total 25(OH)D and 1,25(OH)2 D levels of each participant.

## Conclusion

Our data suggest that vitamin D deficiency may play major role in progression of HDP. Circulatory cytokine/chemokine levels were altered in the HDP group. Furthermore, our analyses suggest that lower vitamin D metabolites were associated with altered cytokines/chemokines and metabolic hormones in HDP. Eotaxin, MCP-1, 25(OH)D, and 1,25(OH)_2_D levels were predictors of HDP as compared to the HP group. However, future studies with large number of subjects are needed to confirm these findings.

## Author Contributions

RA and SKB designed the research; wrote the manuscript. RA measured cytokine/chemokine and metabolic hormone levels; performed data analysis. RB, MB, and RS performed the vitamin D metabolites measurement. BV and NM were clinicians. GV helped in statistical analysis and statistical writing.

## Conflict of Interest Statement

The authors declare that the research was conducted in the absence of any commercial or financial relationships that could be construed as a potential conflict of interest.
