# Supplementary material for: Lower Serum Vitamin D Metabolite Levels in Relation to Circulating Cytokines/Chemokines and Metabolic Hormones in Pregnant Women with Hypertensive Disorders
Source: Front Immunol. 2017 Mar 13;8:273. doi: 10.3389/fimmu.2017.00273 (PMC5346572; doi:10.3389/fimmu.2017.00273)
Supplement: Supplementary file 1 [file data_sheet_1.docx]

**Supplementary file**

**Sample size calculation:** Sample size estimation is based on current prevalence rate of preeclampsia which is 6.7% (Okanlawon, 2015). The nMaster v.2.0 sample size software (http://nmaster.software.informer.com/2.0/) was used to calculate the sample size with 95% power and adjusted ‘α’ that is 2.5% with un-equal allocation group.

**Reference:** Okanlawon. Maternal and perinatal outcome of patients with preeclampsia in a southwest Nigeria. 2015; 32(2):58-64.

**
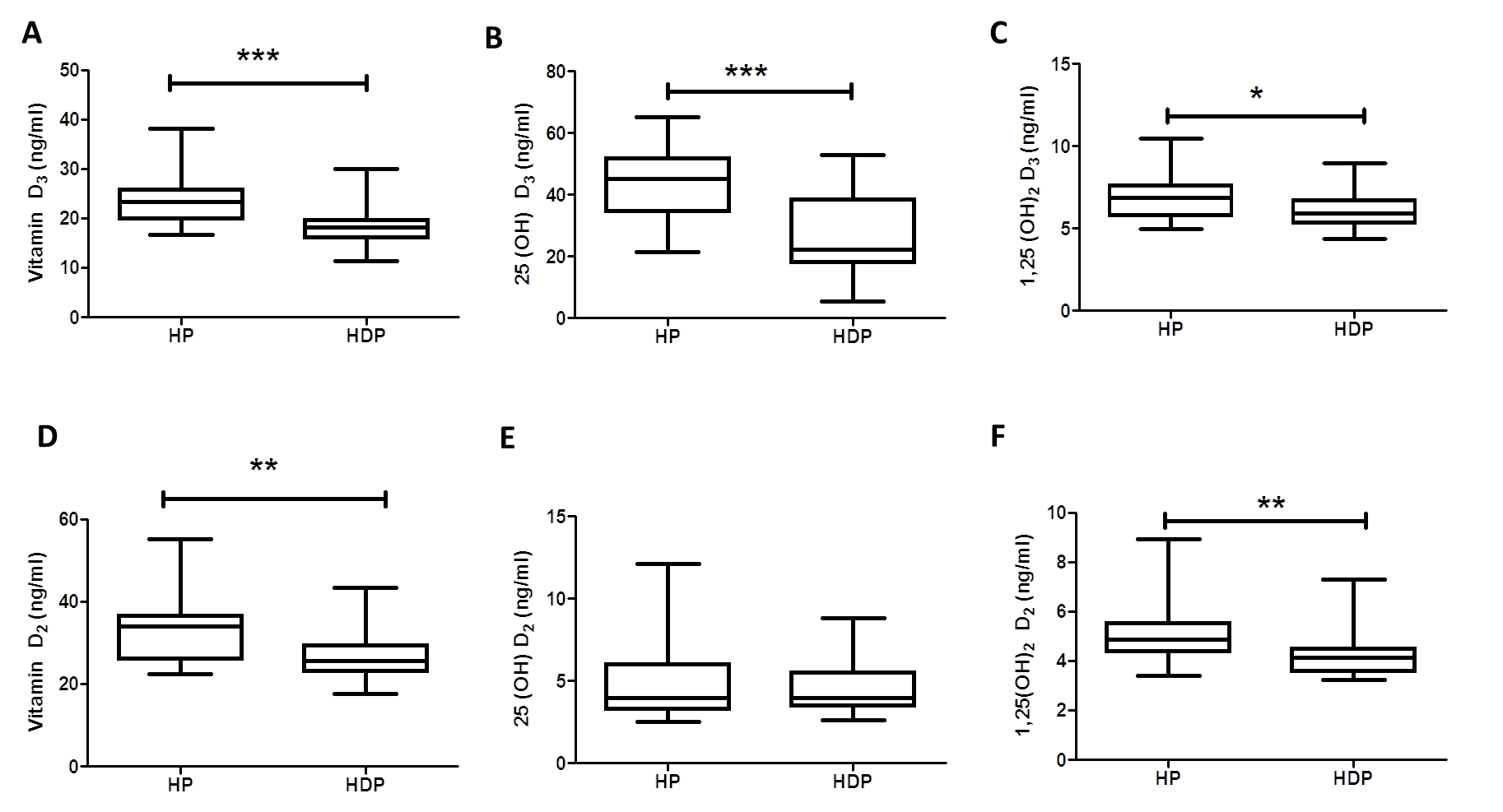
**

**Figure S1:** Vitamin D metabolites A) Vitamin D_3_, B) 25 OH D_3_, C) 1,25 (OH)_2_ D_3_, D) Vitamin D_2_, E) 25 OH D_2_, F) 1,25(OH)_2_ D_2_ levels in combined hypertension disorders in pregnancy (HDP) as compared to healthy pregnancy (HP) group. *p<0.05, **p<0.01, ***p<0.001 vs healthy pregnancy. Mann-Whitney U test was used to determine statistical significance.


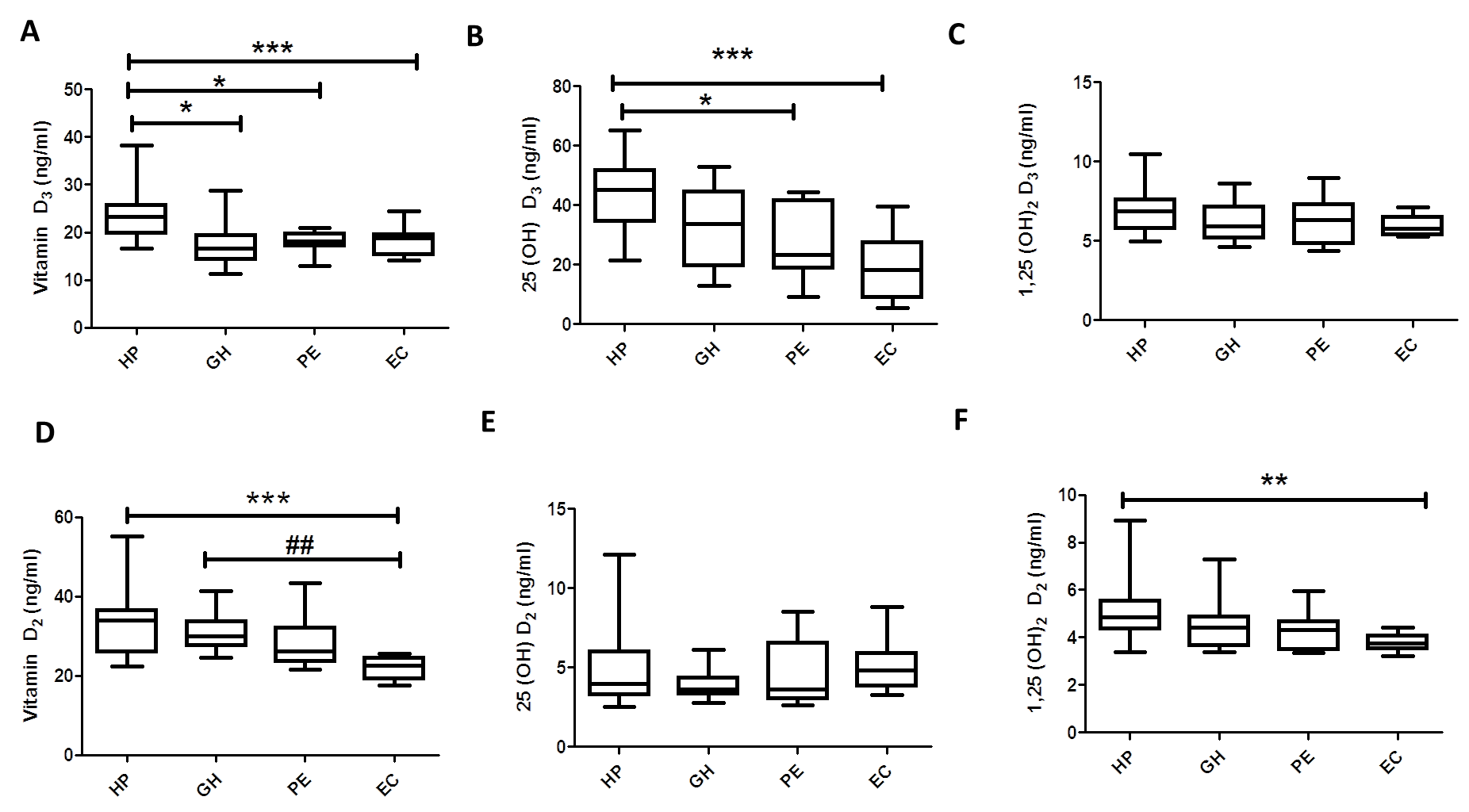


**Figure S2:** Vitamin D metabolites A) Vitamin D_3_, B) 25 OH D_3_, C) 1,25 (OH)_2_ D3, D)Vitamin D_2_, E) 25 OH D_2_, F) 1,25(OH)_2_ D_2_ levels in gestational hypertension (GH), preeclampsia (PE) and eclampsia (EC) as compared to healthy pregnancy (HP) group. *p<0.05, **p<0.01, ***p<0.001 vs healthy pregnancy. ##p<0.01 vs gestational hypertension group. Comparisons between outcome groups Kruskal–Wallis and Dunn’s test.


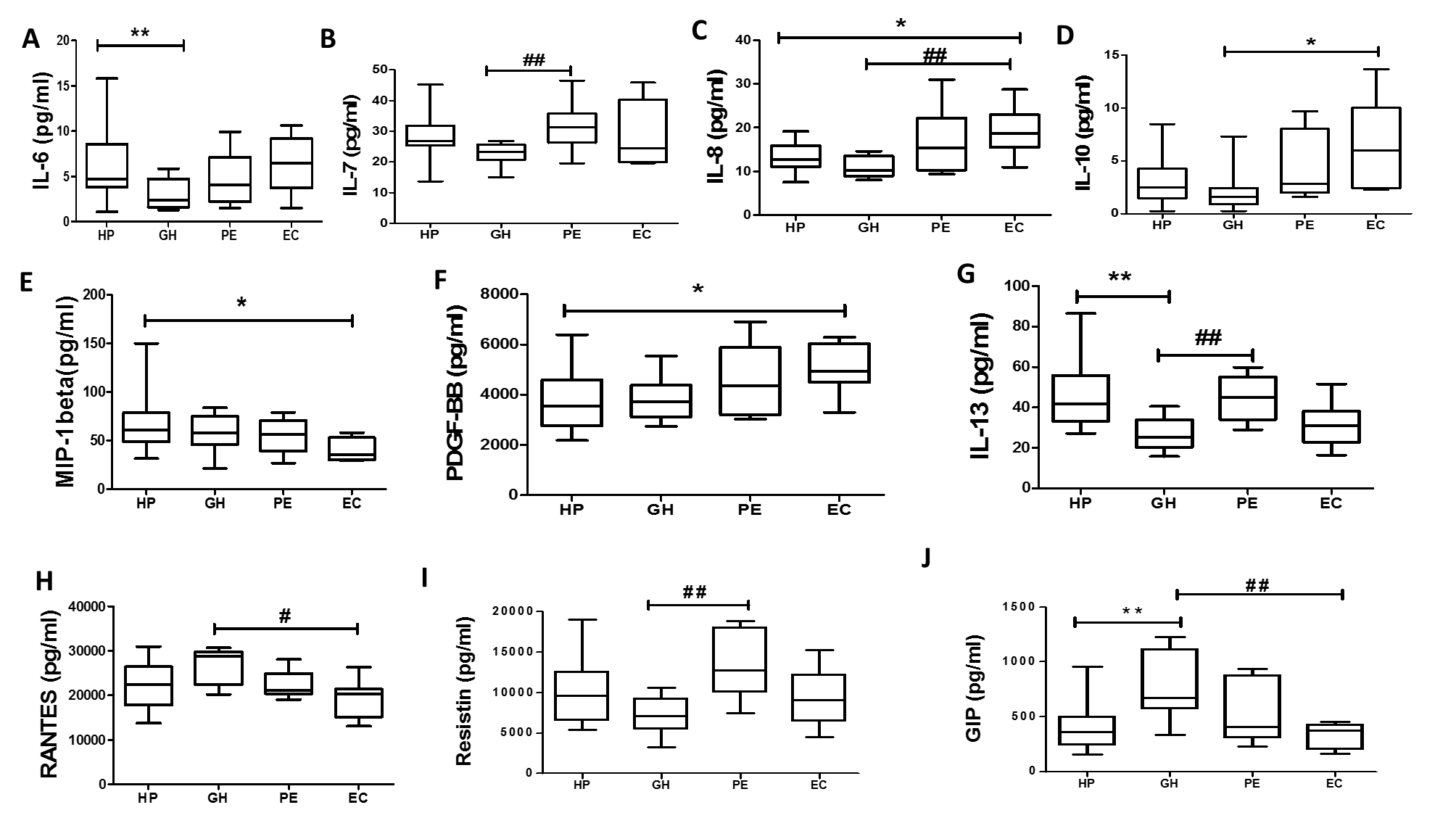
 **Figure S3:** Comparison of significant plasma cytokine/chemokine and metabolic hormones levels in gestational hypertension (GH), preeclampsia (PE), eclampsia (EC) and healthy pregnancy (HP) group. A) IL-6, B) IL-7, C) IL-8, D) IL-10, E) MIP-1beta, F) PDGF-BB, G) IL-13, H) RANTES, I) Resistin, J) GIP. *p<0.05, **p<0.01 vs healthy pregnant; #p<0.05, ##p<0.01 vs gestation hypertension. Comparisons between outcome groups Kruskal–Wallis and Dunn’s test.

**A B**


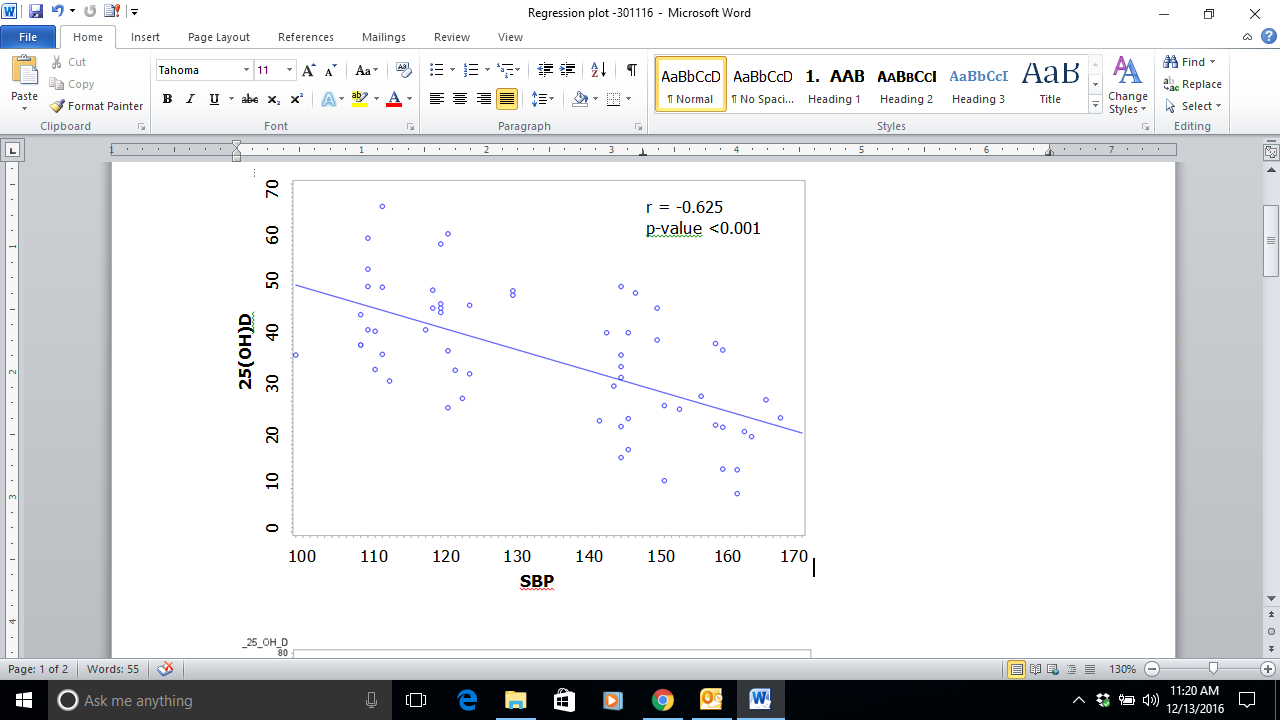

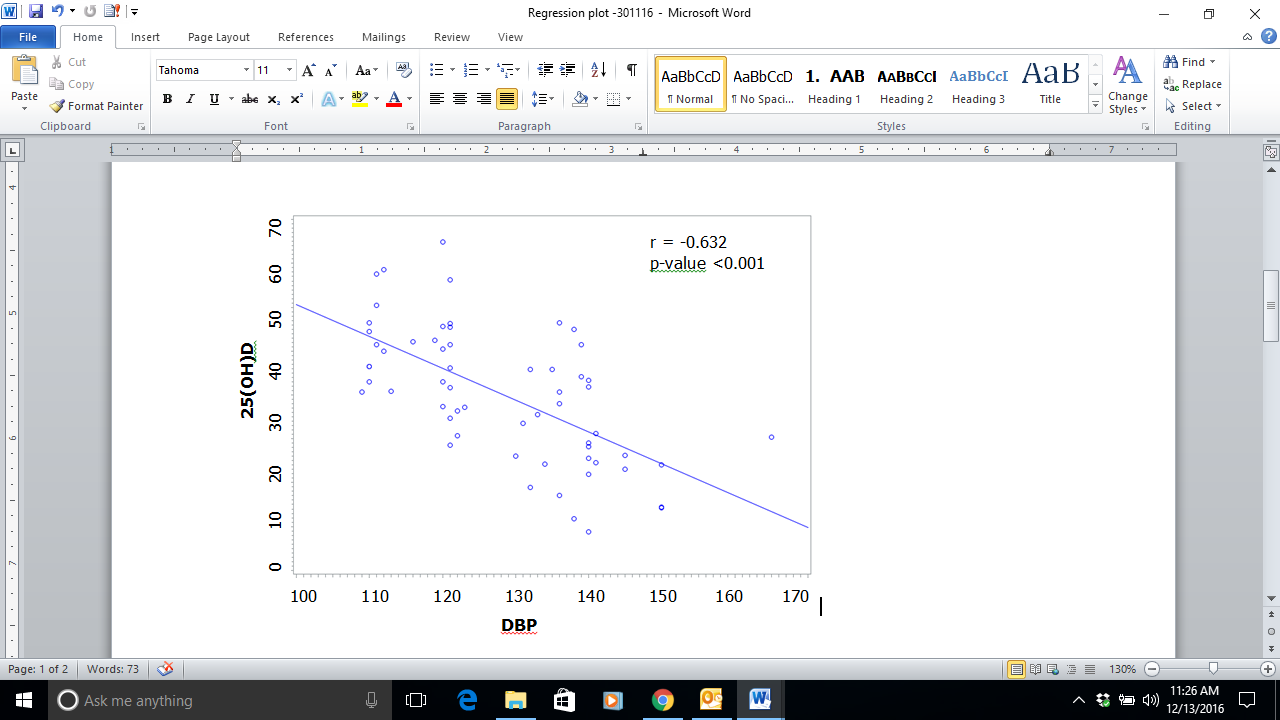


**Figure S4:** The linear relationship between 25 (OH) D ng/ml, systolic blood pressure (SBP) and diastolic blood pressure (DBP) in overall subjects. The correlation between (A) 25 (OH) D and SBP, and (B) 25 (OH) D and DBP.

**A B**


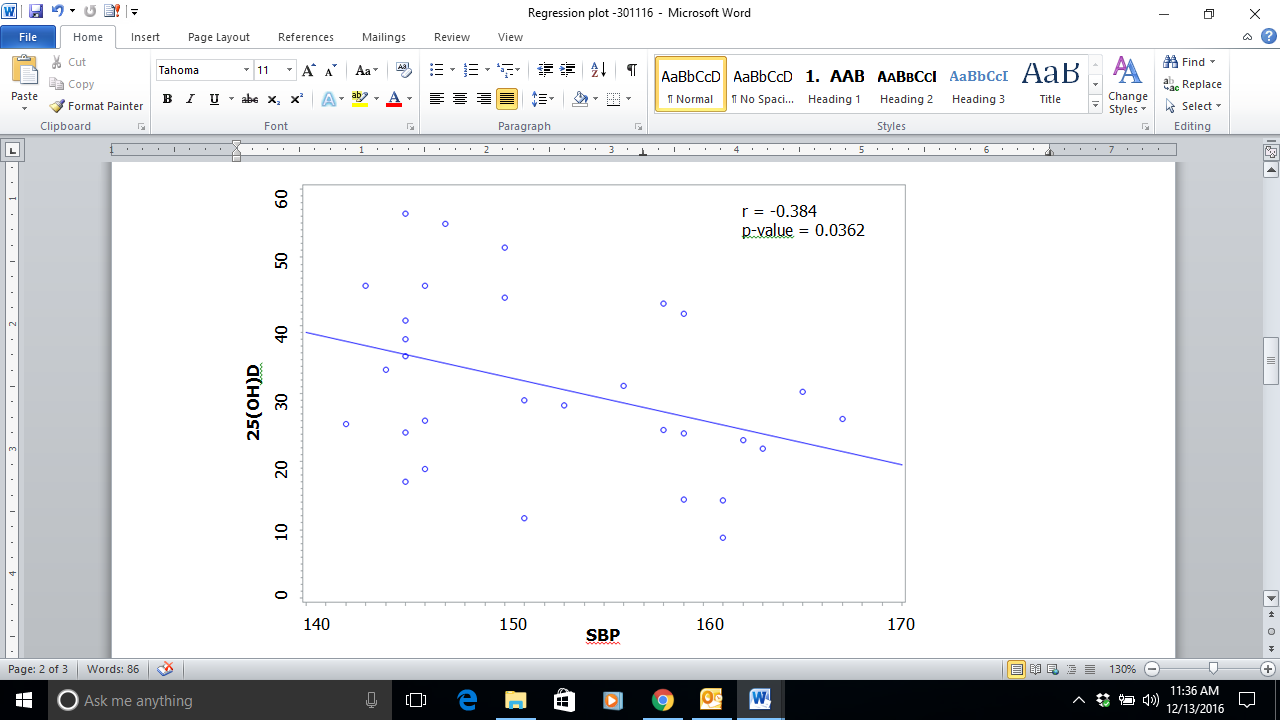

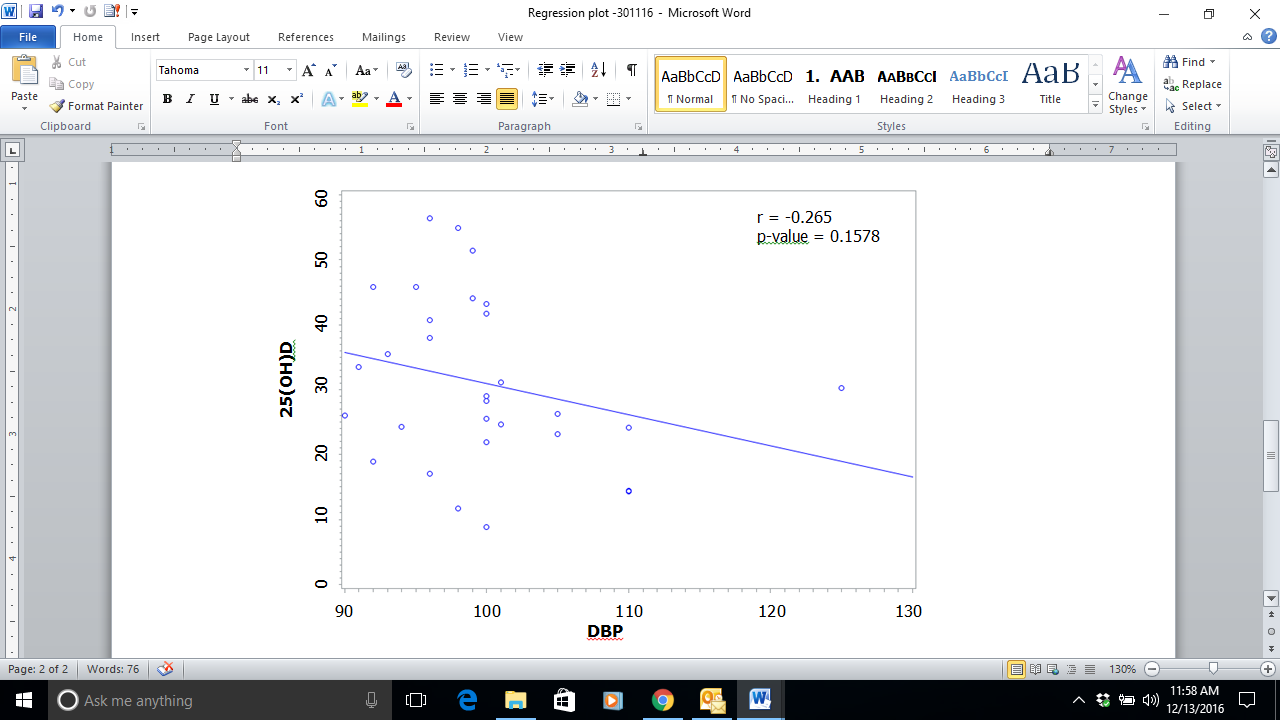


**Figure S5:** The linear relationship between 25 (OH) D ng/ml, systolic blood pressure (SBP) and diastolic blood pressure (DBP) in hypertension disorders subjects. The correlation between (A) 25 (OH) D and SBP, and (B) 25 (OH) D and DBP.

**A B**


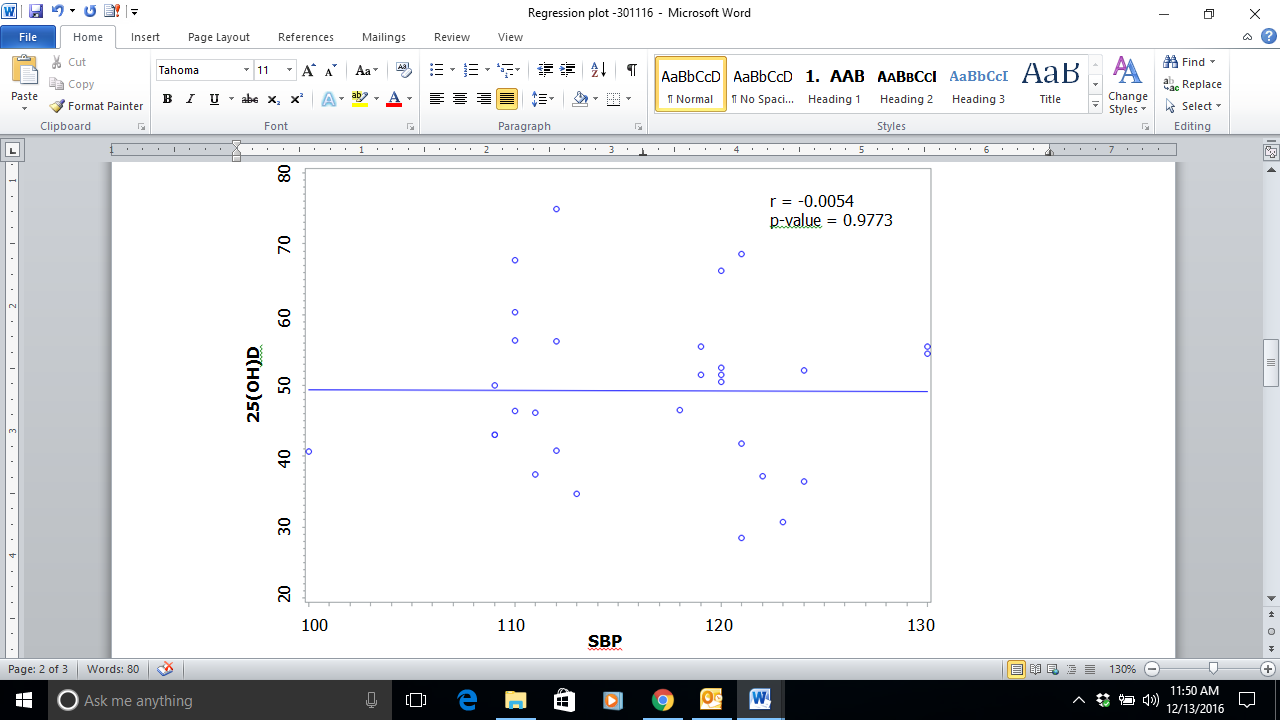

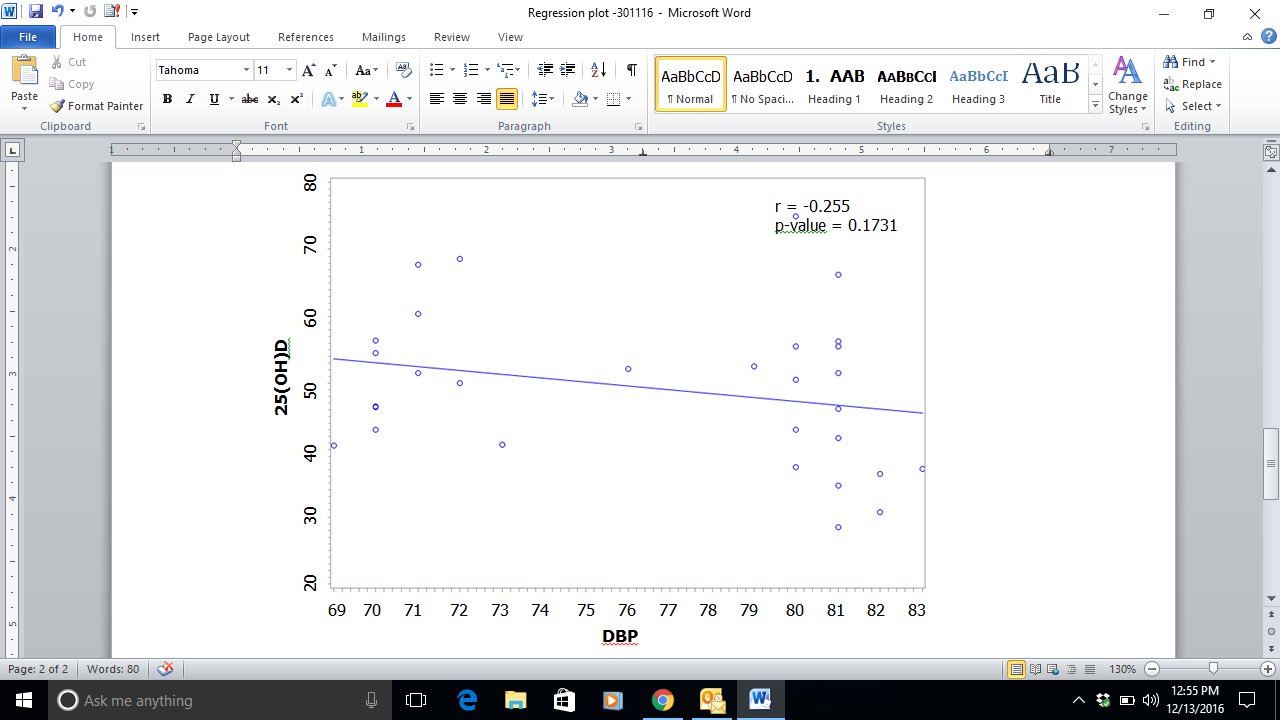


**Figure S6:** The linear relationship between 25 (OH) D ng/ml, systolic blood pressure (SBP) and diastolic blood pressure (DBP) in healthy pregnancy group. The correlation between (A) 25 (OH) D and SBP, and (B) 25 (OH) D and DBP.
